# Arthritis Management: Patient-Reported Health Care Provider Screening, Counseling, and Recommendations for Physical Activity

**DOI:** 10.5888/pcd21.240074

**Published:** 2024-12-19

**Authors:** Elizabeth A. Fallon, Anika L. Foster, Michael A. Boring, David R. Brown, Erica L. Odom

**Affiliations:** 1 Centers for Disease Control and Prevention, Division of Population Health, Atlanta, Georgia; 2 Chenega Services & Federal Solutions, LLC, Chesapeake, Virginia; 3 Centers for Disease Control and Prevention, Division of Nutrition, Physical Activity and Obesity, Atlanta, Georgia

## Abstract

**Introduction:**

Little is known about the recency, correlates, and content of health care provider (HCP) counseling about physical activity (PA) among adults with arthritis.

**Methods:**

We analyzed data from the Porter Novelli FallStyles cross-sectional survey of noninstitutionalized US adults. Among adults with arthritis, we assessed the recency of HCP counseling about PA; counseling content, including PA assessment/screening and advice/counseling; and recommendations. Data were weighted by sex, age, household income, race and ethnicity, household size, education, census region, and metropolitan status.

**Results:**

Among adults with arthritis (n = 1,113), 16.8% received HCP counseling within the past 6 months, 9.6% received counseling between 6 months and a year ago; 27.7% received HCP counseling more than a year ago; 30.4% never received HCP counseling; and 15.5% did not recall. Prevalence of HCP counseling about PA was higher for those reporting obesity (prevalence ratio [PR] = 1.3) and chronic pain (PR = 1.2), compared with those without these conditions. The most and least common content of HCP counseling were assessment of PA level (74.7%) and receiving a physical activity prescription (6.1%), respectively. The most frequent recommendations for PA type were flexibility exercises (40.1%), aerobic activities (39.8%), specific modalities of PA (eg, swimming, walking, dancing; 38.1%), and muscle-strengthening exercises (36.6%). Only 4.4% received a recommendation for arthritis-appropriate PA programs.

**Conclusion:**

HCP counseling about PA among adults with arthritis for arthritis symptom management is lacking in frequency, actionable content, and recommendations to engage in evidence-based PA interventions. Dissemination and implementation of policies and programs facilitating frequent high-quality HCP counseling and recommendation to PA programs for arthritis remains a public health priority.

**Summary**

**What is already known on this topic?**

Health care provider (HCP) counseling is effective for improving physical activity (PA) among older adults, and evidence shows that regular PA improves arthritis pain and physical function.

**What is added by this report?**

In fall of 2020, 54% of adults with arthritis reported ever receiving HCP counseling about PA to manage arthritis symptoms, and the rates were higher among adults reporting obesity and chronic pain compared with those without these conditions. The most common patient–provider interactions included assessment of PA level and counseling about activity type. Only 4% received recommendations for arthritis-appropriate evidence-based interventions.

**What are the implications for public health practice?**

Facilitating HCP counseling for evidence-based PA programs among adults with arthritis remains a public health priority.

## Introduction

Arthritis is a leading cause of chronic pain, disability, and dispensed opioid prescriptions (1–3). Based on research showing that regular physical activity (PA) improves arthritis pain and physical function, PA is strongly or conditionally recommended by the American College of Rheumatology for osteoarthritis (4), rheumatoid arthritis (5), and psoriatic arthritis (6). Additionally, emerging research shows benefits of PA for gout and fibromyalgia (7–9). The 2018 Physical Activity Guidelines for Americans (10) recommend that all adults, including those with any form of arthritis, engage in at least 150 minutes per week of moderate-intensity aerobic PA (or 75 minutes of vigorous-intensity or an equivalent combination of moderate- to vigorous-intensity aerobic PA), and 2 days a week of muscle-strengthening PA. Older adults (aged ≥65 y) also need multicomponent PA comprising balance training and aerobic and muscle-strengthening activities (10). However, adults with arthritis have lower prevalence of meeting the aerobic and strength training guidelines, compared with adults without arthritis (11,12).

Health care provider (HCP) screening, counseling, and recommendation for PA has a small to medium effect size for increasing PA among adults (13,14). Because more than 80% of US adults report at least 1 doctor visit annually (15), routine HCP counseling and recommendation for PA could have a substantial public health impact for adults with arthritis. Thus, increasing the proportion of adults with arthritis receiving HCP counseling and recommendation for PA is a public health priority (16,17). Previous research exploring HCP self-reported behavior found that 49.2% always assess PA level, 57.7% always recommend PA, and 39.7% engage in both assessment and recommendation of PA for adults with arthritis (18). Less is known about the correlates of receiving HCP counseling about PA among adults with arthritis, or the recency and content of HCP counseling from the perspective of adults with arthritis. Therefore, this study will 1) describe the proportion of adults with arthritis receiving HCP counseling about PA as an arthritis management strategy, 2) identify factors associated with receiving HCP counseling about PA, and 3) describe the content of HCP counseling when counseling was received.

## Methods

### Design and sample

We analyzed cross-sectional data from the Porter Novelli FallStyles survey collected through the Ipsos KnowledgePanel (Ipsos Group SA) from September 24, 2020, to October 10, 2020 (19). KnowledgePanel participants are recruited by mail using probability-based sampling by address, which produces a sample representative of the noninstitutionalized US population. If needed, people were provided access to a laptop or tablet and access to the internet to participate in online surveys. The online survey invitation was sent to 4,548 panelists aged 18 years or older. Participants were informed that they were participating in market research, were not required to answer any item, and were able to exit the survey at any time. Three reminders were sent to nonresponders to increase response rates. As incentive for participating, survey participants received 5,000 points (equivalent to $5) which can be redeemed for gift cards and prizes. A person who failed to answer at least half of the survey questions or complete the survey in less than 5 minutes was considered by Ipsos to have an incomplete survey and to be a nonresponder when calculating the response rate. The response rate was 79.7% (3,625 of 4,548). Data were weighted by sex, age, household income, race and ethnicity, household size, education, census region, and metropolitan status to match US population estimates based on the 2019 US Current Population Survey. Because deidentified survey data from Porter Novelli were used, this research was considered exempt from review by the Centers for Disease Control and Prevention’s (CDC’s) institutional review board.

The Figure shows the sample selection process and sample size for the following 3 analyses: 1) descriptive statistics of adults with arthritis in the sample and the proportion reporting receiving HCP counseling about PA, 2) logistic regression exploring correlates of receiving HCP counseling about PA, and 3) descriptive statistics for HCP PA counseling content. Survey participants (n = 3,625) were excluded from the first analysis if they had missing data for the items assessing doctor-diagnosed arthritis (n = 16), did not have doctor-diagnosed arthritis (n = 2,495), or were missing data for the item assessing receipt of HCP counseling about PA (n = 1). The final sample size for the first analysis was 1,113. Only participants included in the first analysis met inclusion criteria for the second and third analyses. Participants were excluded from the second analysis if they did not recall whether they received any HCP counseling about PA (n = 175) or had missing data for any variable included in the multivariable logistic regression. Participants were excluded for the third analysis if they did not recall whether they received any HCP counseling about PA (n = 175), did not receive any HCP counseling about PA (n = 346), or had missing data for survey questions about HCP counseling content.

**Figure:**
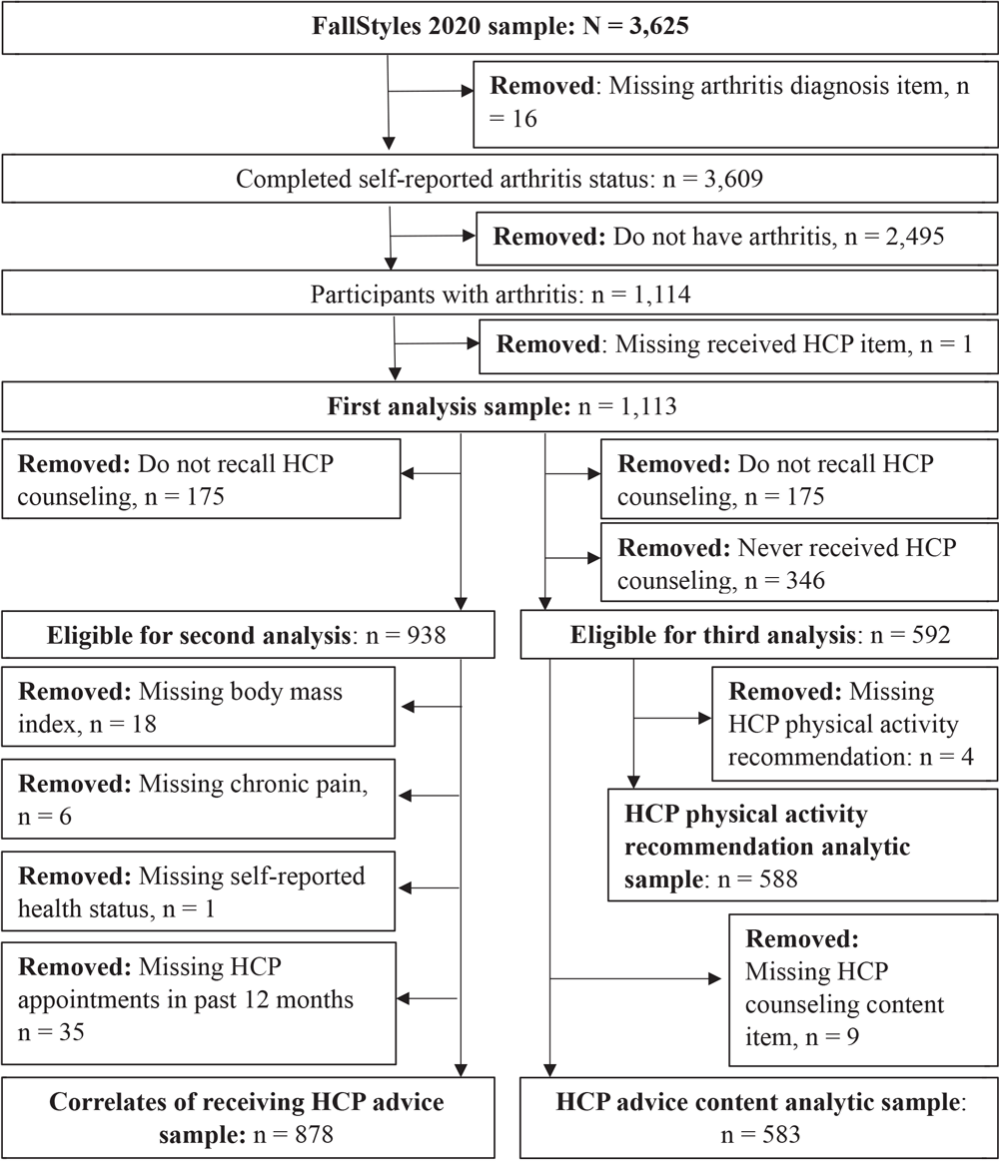
Flow diagram of the inclusion and exclusion criteria, FallStyles Survey (September 24, 2020, to October 10, 2020), including sample sizes for each phase of the analysis. Abbreviation: HCP, health care provider.

### Measures

Doctor-diagnosed arthritis status was assessed by asking “Have you ever been told by a doctor or other health professional that you have some form of arthritis, rheumatoid arthritis, gout, lupus, or fibromyalgia?” with response options of yes or no. Sociodemographic variables included self-reported sex, age, race and ethnicity, and highest educational attainment. Self-reported height and weight were used to calculate body mass index (BMI, weight in kg divided by height in m^2^) and then collapsed into 3 categories (underweight/healthy weight, <25; overweight, 25 to <30; obesity, ≥30). Participants were also asked “During the past year, have you had (or do you currently have) any of these health conditions? Select all that apply.” The following 9 chronic health conditions were included in this study: chronic pain, asthma, diabetes, emphysema/chronic obstructive pulmonary disease (COPD), high blood pressure, atrial fibrillation/congestive heart failure/other heart condition (angina or heart attack), stroke, other cancer (skin cancer was listed immediately before other cancer and not included in this study), or depression. Due to their relevance for arthritis outcomes and PA, chronic pain and depression were coded individually (selected/not selected). The remaining 7 physical health conditions were summed for each participant, and then collapsed into 3 groups (none, 1, 2 or more). Self-reported health status was assessed with the item “In general, would you say your health is . . . ?” with the following response options: excellent, very good, good, fair, and poor. These responses were then collapsed into 3 groups (very good/excellent, good, fair/poor). Finally, health care provider visits in the past 12 months were assessed by asking “How many times in the past 12 months have you visited each of the following kinds of health professionals for your own health care? (If the answer is none, type in ‘0’),” followed by “primary care doctors (eg, family practitioner, internist, OB/GYNs),” and “specialist doctors (eg, cardiologist, oncologist, dermatologist)”; responses were summed and 5 categories were created (0, 1, 2, 3, and 4 or more).

Three survey items assessed HCP assessment and screening, counseling, and recommendations for PA. Participants were first asked “Has a doctor or other health professional ever suggested PA or exercise to help your arthritis or joint symptoms?” with response options “Yes, within the last 6 months,” “Yes, between 6 months and a year ago,” “Yes, more than a year ago,” “No, never,” and “I do not recall.” Those responding with any of the 3 “Yes” options to the initial question were subsequently asked “When your doctor or health care provider talked to you about PA or exercise, which of the following did they do? Select all that apply.” Response options included 1) ask you about your current level of PA/exercise; 2) give you handouts or other information about PA/exercise; 3) write you a prescription for PA/exercise; 4) ask you about your barriers to, or what gets in the way of, PA/exercise; 5) talk to you about how to overcome these barriers to PA/exercise; 6) talk to you about how much PA you should be getting or doing; 7) talk to you about the types of PA you should be getting or doing; and 8) something else not listed.

Finally, participants were asked “Which, if any, of the following has your doctor or health care provider ever recommended? Select all that apply” with response options including the following: 1) aerobic activities that increase your heart rate like brisk walking, cycling, swimming, water aerobics; 2) muscle-strengthening exercises like lifting weights, working with resistance bands, or yoga; 3) flexibility exercises like stretching and yoga; 4) balance exercises like walking backward, standing on one foot, and tai chi; 5) group exercise classes or programs in general; 6) group exercise classes or programs to manage arthritis; 7) specific forms of PA like swimming, walking, or dancing; and 8) none of these.

### Analysis

Descriptive statistics by demographic and health characteristics were calculated to describe adults with arthritis in the sample. Additionally, participants’ responses to if or when HCP counseling about PA was received were described. Subsequently, prevalence ratios (PRs) comparing the proportion of adults with arthritis receiving HCP advice for PA across multiple demographic and health characteristics were obtained by using multivariable logistic regression. Finally, crude counts and weighted prevalence of HCP counseling and recommendation content were calculated. All analyses were conducted by using SAS version 15.2 (SAS Institute, Inc) and included sampling weights.

## Results

Participants included in the study were adults with arthritis who were primarily aged 60 years or older (57.5%), were non-Hispanic White (67.9%), rated their health as good (42.0%) or very good/excellent (33.9%), and had zero (37.1%) or one (35.7%) physical health conditions other than arthritis (Table 1). Regarding when their HCP recommended PA to manage arthritis or joint symptoms, 16.8% of adults with arthritis reported it was within the last 6 months, 9.6% reported it was between 6 months and 1 year ago, and 27.7% reported it was more than 1 year ago. The sum of these 3 categories resulted in over half (54.1%) of adults with arthritis reporting they ever-received HCP counseling for PA. Many adults with arthritis reported their HCP never suggested PA to manage arthritis/joint symptoms (30.4%) and 15.5% did not recall (Table 2).

**Table 1:** US Adults With Arthritis^a^, by Demographic and Health Characteristics, FallStyles Survey, September 24, 2020–October 10, 2020^b^

Characteristic	Total sample (N = 1,113)	
	Crude count^c^	Weighted^d^ % (95% CI)
**Sex**		
Male	493	42.1 (38.7–45.4)
Female	620	57.9 (54.6–61.3)
**Age group, y**		
18–44	101	15.6 (12.5–18.7)
45–59	257	26.9 (23.8–30.0)
≥60	755	57.5 (54.0–61.0)
**Race and ethnicity**		
Non-Hispanic White	864	67.9 (64.3–71.4)
Non-Hispanic Black	86	11.5 (9.1–13.9)
Hispanic, Non-Hispanic Other, ≥2 races	163	20.6 (17.4–23.9)
**Education**		
Less than or equal to high school degree/high school equivalence	415	42.2 (38.8–45.6)
Some college	327	30.4 (27.3–33.5)
Bachelor’s degree or higher	371	27.4 (24.6–30.1)
**Obesity status (BMI, kg/m^2^)**		
Underweight/healthy weight (<25)	241	22.2 (19.3–25.1)
Overweight (25 to <30)	356	31.8 (28.7–35.0)
Obesity (≥30)	491	45.9 (42.5–49.3)
**Chronic pain** e		
No	753	66.6 (63.4–69.9)
Yes	353	33.4 (30.1–36.6)
**Physical health condition(s)** e		
None	401	37.1 (33.8–40.4)
1	401	35.7 (32.4–38.9)
≥2	304	27.2 (24.2–30.2)
**Depression** e		
No	896	78.0 (75.0–81.0)
Yes	210	22.0 (19.0–25.0)
**Self-reported health status**		
Very good/excellent	405	33.9 (30.7–37.0)
Good	466	42.0 (38.7–45.3)
Fair/poor	241	24.1 (21.1–27.1)
**Health care provider visits in past 12 months^f^**		
0	57	6.0 (4.2–7.8)
1	121	11.6 (9.3–14.0)
2	173	15.8 (13.4–18.2)
3	159	15.2 (12.7–17.7)
≥4	562	51.3 (47.9–54.7)

Abbreviations: BMI, body mass index; COPD, chronic obstructive pulmonary disease; OB/GYN, obstectrics/gynecology.

a Participants answering yes to the item “Have you ever been told by a doctor or other health professional that you have some form of arthritis, rheumatoid arthritis, gout, lupus, or fibromyalgia?” were considered to have arthritis.

b The Porter Novelli FallStyles 2020 survey was collected in the US among adults aged 18 years or older.

c Some columns may not sum to 1,113 due to missing data for that item.

d Data were weighted by sex, age, household income, race and ethnicity, household size, education, census region, and metropolitan status.

e Assessed by using the item “During the past year, have you had (or do you currently have) any of these health conditions? Select all that apply” with response options of depression, chronic pain, asthma, diabetes, emphysema/COPD, high blood pressure, atrial fibrillation/congestive heart failure/other heart condition (angina or heart attack), stroke, or cancer (not including skin cancer). Due to their relevance to arthritis outcomes and physical activity, depression and chronic pain were coded individually (selected/not selected). The remaining physical health conditions were summed for each participant and then categorized into 3 groups (none, 1, 2 or more).

f Health care provider visits in the past 12 months was assessed by asking “How many times in the past 12 months have you visited each of the following kinds of health professionals for your own health care? (If the answer is none, type in ‘0’),” followed by “primary care doctors (eg, family practitioner, internist, OB/GYNs),” and “specialist doctors (eg, cardiologist, oncologist, dermatologist)”; responses were summed and 5 categories were created (0, 1, 2, 3, and 4 or more.

**Table 2:** Patient-Reported Receipt of Health Care Provider Counseling About Physical Activity^a^ Among US Adults With Arthritis^b^, FallStyles Survey, September 24, 2020–October 10, 2020^c^

Patient-reported receipt of health care provider counseling about physical activity	Total sample (N = 1,113)	
	Crude count	Weighted^d^ % (95% CI)
Yes, within the last 6 months	182	16.8 (14.3–19.3)
Yes, between 6 months and 1 year ago	104	9.6 (7.5–11.6)
Yes, more than 1 year ago	306	27.7 (24.6–30.7)
No, never	346	30.4 (27.3–33.5)
I do not recall	175	15.5 (13.1–17.9)

a Participants were asked “Has a doctor or other health professional ever suggested physical activity or exercise to help your arthritis or joint symptoms?” with the following response options: “Yes, within the last 6 months,” “Yes, between 6 months and a year ago,” “Yes, more than a year ago,” “No, never,” and “I do not recall.”

b Participants answering yes to the item “Have you ever been told by a doctor or other health professional that you have some form of arthritis, rheumatoid arthritis, gout, lupus, or fibromyalgia?” were considered to have arthritis.

c The Porter Novelli FallStyles 2020 survey was collected in the US among adults aged 18 years or older.

d Data were weighted by sex, age, household income, race and ethnicity, household size, education, census region, and metropolitan status.

After controlling for all other variables in the multivariable analysis, obesity and chronic pain were the only characteristics significantly correlated with ever receiving HCP counseling about PA (Table 3). Specifically, compared with adults with arthritis reporting a BMI <25, those who reported obesity (BMI ≥30) had higher prevalence of receiving HCP counseling about PA (PR = 1.30). Additionally, compared with adults with arthritis not reporting chronic pain, those who reported chronic pain had higher prevalence of receiving HCP counseling about PA (PR = 1.24).

**Table 3:** Correlates of Ever Receiving Health Care Provider Counseling About Physical Activity^a^ Among US Adults With Arthritis^b^, FallStyles Survey, September 24, 2020–October 10, 2020^c^

Characteristic	Analytic sample (n = 878)	
	% (95% CI)	Prevalence ratio (95% CI)^d^
**Sex**		
Male [Reference]	60.6 (55.0–65.9)	—
Female	66.5 (61.7–71.0)	1.12 (1.00–1.25)
**Age group, y**		
18–44 [Reference]	60.6 (55.0–65.9)	—
45–59	66.1 (58.6–72.9)	1.07 (0.85–1.35)
≥60	64.0 (59.9–67.9)	1.04 (0.83–1.29)
**Race/ethnicity**		
NH White [Reference]	62.3 (58.4–66.1)	—
NH Black	70.9 (58.3–80.9)	1.05 (0.87–1.27)
Hispanic, NH other, ≥2 races	65.5 (55.5–74.2)	1.09 (0.94–1.27)
**Education**		
Less than or equal to high school degree/high school equivalence	65.6 (59.6–71.1)	1.03 (0.90–1.19)
Some college	66.8 (59.9–73.1)	1.09 (0.96–1.24)
Bachelor’s degree or higher [Reference]	58.7 (52.8–64.4)	—
**Obesity status (BMI, kg/m^2^)**		
Underweight/healthy weight (<25) [Reference]	51.3 (43.3–59.2)	—
Overweight (25 to <30)	61.7 (55.3–67.8)	1.20 (0.99–1.45)
Obesity (≥30)	72.0 (66.7–76.8)	1.30 (1.08–1.55)
**Chronic pain**		
No [Reference]	58.4 (54.0–62.8)	—
Yes	75.2 (69.0–80.4)	1.24 (1.10–1.40)
**Physical health condition(s)^e^**		
None [Reference]	56.6 (50.5–62.5)	—
1	65.0 (59.0–70.5)	1.03 (0.89–1.19)
≥2	73.2 (66.5–79.0)	1.13 (0.98–1.31)
**Depression**		
No [Reference]	62.4 (58.5–66.2)	—
Yes	70.2 (61.3–77.8)	0.96 (0.81–1.13)
**General health**		
Very good/excellent [Reference]	54.4 (48.4–60.3)	—
Good	66.2 (60.7–71.3)	1.08 (0.95–1.24)
Poor/fair	73.9 (66.1–80.5)	1.10 (0.92–1.31)
**Health care provider visits in past 12 months^f^**		
0 [Reference]	49.9 (33.6–66.2)	—
1	52.5 (40.4–64.3)	1.10 (0.77–1.58)
2	54.3 (45.5–62.9)	1.12 (0.81–1.54)
3	70.7 (60.2–79.4)	1.31 (0.95–1.81)
≥4	69.3 (64.5–73.8)	1.25 (0.92–1.69)

Abbreviations: BMI, body mass index; NH, non-Hispanic.

a Assessed by using the item “Has a doctor or other health professional ever suggested physical activity or exercise to help your arthritis or joint symptoms?” with the following response options: “Yes, within the last 6 months,” “Yes, between 6 months and a year ago,” “Yes, more than a year ago,” “No, never,” and “I do not recall.” Individuals answering any of the 3 yes responses (n = 592) were included in this analysis.

b Participants answering yes to the item “Have you ever been told by a doctor or other health professional that you have some form of arthritis, rheumatoid arthritis, gout, lupus, or fibromyalgia?” were considered to have arthritis.

c The Porter Novelli FallStyles 2020 survey was collected in the US among adults aged 18 years or older.

d Prevalence ratios were obtained by using a multivariable logistic regression including all variables listed in the table. Data were weighted for sex, age, household income, race and ethnicity, household size, education, census region, and metropolitan status.

e Assessed by using the item “During the past year, have you had (or do you currently have) any of these health conditions? Select all that apply” with response options including depression, chronic pain, asthma, diabetes, emphysema/COPD, high blood pressure, atrial fibrillation/congestive heart failure/other heart condition (angina or heart attack), stroke, or cancer (not including skin cancer). Due to their relevance to arthritis outcomes and physical activity, depression and chronic pain were coded individually (selected/not selected). The remaining physical health conditions were summed for each participant, and then categorized into 3 groups (none, 1, 2 or more).

f Health care provider visits in the past 12 months was assessed by asking “How many times in the past 12 months have you visited each of the following kinds of health professionals for your own health care? (If the answer is none, type in ‘0’),” followed by “primary care doctors (eg, family practitioner, internist, OB/GYNs),” and “specialist doctors (eg, cardiologist, oncologist, dermatologist)”; responses were summed and 5 categories were created (0, 1, 2, 3, and 4 or more).

Among adults with arthritis reporting their HCP ever suggested PA to manage arthritis or joint symptoms (Table 4), 74.7% were asked about their PA level, 50.9% were told about the types of PA they should be doing, and 44.4% were told about the amounts of PA they should be doing. Few received handouts (29.0%), less than 1 in 4 talked with their HCP about PA barriers (22.8%) or talked with their HCP about overcoming barriers (16.4%). Only 6.1% reported receiving a PA prescription.

**Table 4:** Patient-Reported Health Care Provider Counseling Content Among US Adults With Arthritis^a^ Ever-Receiving Health Care Provider Counseling About Physical Activity^b^, FallStyles Survey, September 24, 2020–October 10, 2020^c^

Item/question	Crude count	Weighted % (95% CI)^d^
**When your health care provider talked about physical activity, which of the following did they do? (n = 583)** e ^,^ f		
Ask about current physical activity level	454	74.7 (70.3–79.0)
Talk about the types of physical activity should be doing	297	50.9 (46.2–55.5)
Talk about how much physical activity should be getting	261	44.4 (39.8–49.0)
Give handouts or information	155	29.0 (24.7–33.3)
Ask about physical activity barriers	136	22.8 (19.0–26.6)
Talk about how to overcome barriers	86	16.4 (12.6–20.2)
Something else	36	7.2 (4.5–10.0)
Physical activity prescription	33	6.1 (3.5–8.6)
**Which, if any, of the following has your health care provider ever recommended? (n = 588)** e ^,^ f		
Flexibility exercises (eg, stretching, yoga)	244	40.1 (35.6–44.6)
Aerobic activities (eg, brisk walking, cycling, swimming, water aerobics)	238	39.8 (35.2–44.3)
Specific forms of physical activity (eg, swimming, walking, dancing)	222	38.1 (33.6–42.7)
Muscle-strengthening exercises (eg, lifting weights, resistance bands, yoga)	222	36.6 (32.1–41.1)
Balance exercises (eg, walking backward, standing on one foot, tai chi)	127	21.1 (17.3–24.9)
Group exercise classes or programs, in general	41	7.0 (4.3–9.7)
Group exercise classes or programs to manage arthritis	28	4.4 (2.6–6.2)
None of these	92	15.8 (12.4–19.2)

a Participants answering yes to the item “Have you ever been told by a doctor or other health professional that you have some form of arthritis, rheumatoid arthritis, gout, lupus, or fibromyalgia?” were considered to have arthritis.

b Assessed by using the item “Has a doctor or other health professional ever suggested physical activity or exercise to help your arthritis or joint symptoms?” with the following response options: “Yes, within the last 6 months,” “Yes, between 6 months and a year ago,” “Yes, more than a year ago,” “No, never,” and “I do not recall.” Individuals answering any of the 3 yes responses (n = 592) were included in this analysis.

c The Porter Novelli FallStyles 2020 survey was collected in the US among adults aged 18 years or older.

d Data were weighted by sex, age, household income, race and ethnicity, household size, education, census region, and metropolitan status.

e Sample is smaller than the number eligible due to missing data for the item.

f A list of responses to this item were provided, and participants were allowed to select all that apply. Response options are presented based on frequency of selection in descending order and do not reflect the order in which the responses were presented to participants.

Regarding recommendations for PA type (Table 4), flexibility exercises (40.1%), aerobic activities (39.8%), specific forms of PA (38.1%), and muscle-strengthening exercises (36.6%) were the most recommended. HCPs rarely recommended adults with arthritis to group exercise classes or PA programs for arthritis management (4.4%), and 15.8% of adults with arthritis reported no HCP counseling about PA type.

## Discussion

About half (54.1%) of adults with arthritis in this study reported ever receiving HCP counseling about PA for arthritis management. This was similar to the 52.1% estimated by Healthy People 2030 using 2019 National Health Interview Survey data (17) but lower than the median state prevalence of HCP counseling about PA (70.4%) reported from the 2019 Behavioral Risk Factor Surveillance System (20). Prevalence differences may be due to a combination of methodologic differences including sample size and composition, recruitment method, survey delivery (eg, online vs telephone-based), weighting methods, how items measuring patient-reported HCP advice for PA are worded, and contextual differences in items preceding the question assessing HCP counseling about PA. Additionally, previous research shows that adults with a higher number of self-reported comorbidities are more likely to receive HCP for PA (21). Most of the adults in this study reported being in good or very good/excellent health, with zero or 1 physical chronic condition in addition to arthritis, which may have resulted in lower rates of HCP counseling about PA compared with other recent national US surveys (17,20).

After controlling for other factors, obesity and chronic pain status were associated with receiving HCP counseling about PA to manage arthritis. This is consistent with 2019 Behavioral Risk Factor Surveillance System data showing that adults with obesity reported higher prevalence of HCP counseling about PA to manage arthritis (74.5%) compared with adults with BMI in the overweight (69.4%) or underweight/healthy weight (66.9%) ranges (20). Duca and colleagues (20) also showed that prevalence of receiving HCP counseling about PA to manage arthritis was higher among adults with arthritis reporting severe joint pain (74.5%), compared with those reporting no/mild joint pain (66.3%).

Our findings showed that most HCP counseling content is assessing amount of PA, with only about half explaining which types of PA adults with arthritis should be doing. When HCPs counseled for the type of PA adults with arthritis should be doing, they most frequently recommended flexibility, aerobic, and muscle-strengthening activities, consistent with the national PA guidelines (10). However, less than 1 in 4 adults with arthritis were asked about barriers (22.8%) or received counseling about PA barriers (16.4%), less than 1 in 10 adults with arthritis (7%) in this study were recommended to specific group exercise classes or community-based programs, and less than 1 in 20 (4.4%) were recommended to CDC-recognized, arthritis-appropriate PA programs. These results are consistent with previous research exploring HCP self-reported PA counseling content using Porter Novelli’s DocStyles 2018 online survey. Specifically, Guglielmo et al found that HCPs most frequently recommended aerobic (eg, walking, swimming, or cycling) and stretching/flexibility activities to their patients with arthritis and had low rates of recommending community-based PA programs/classes, or arthritis-appropriate programs/classes (22). Furthermore, Guglielmo et al found that lack of HCP awareness of community-based arthritis-appropriate programs may be a contributing factor for lack of recommendations (22).

Our study has at least 6 limitations. First, it is subject to selection bias, which may have affected estimates. The address-based sampling did not include people without housing or those housed in prisons or medical facilities, and as noted, the adults in this study had good self-rated health and low prevalence of additional chronic disease beyond arthritis. Additionally, estimates may be biased if members of the Ipsos KnowledgePanel who opted in to this particular survey were different than members who chose not to participate. Second, our study relies on self-reported information from the patients’ perspective, which is subject to self-presentation and recall biases. Third, this study was conducted during the COVID-19 pandemic, and adults with chronic disease may not have been attending HCP appointments as frequently or in person during this time, which may have influenced actual or perceived HCP counseling about PA. However, it is noteworthy that few adults with arthritis reported attending no HCP appointments in the preceding 12 months. Additionally, the COVID-19 pandemic resulted in pausing or ending in-person community-based PA programs, potentially lowering HCP ability or willingness to recommend these programs to their patients, especially patients at high risk of COVID-19 illness and death. Fourth, the survey items were closed-ended and did not allow participants to provide context to their responses; therefore, this study may not represent the full spectrum of arthritis patients’ experiences with HCP counseling about PA. Fifth, the item assessing recency of HCP advice for PA may have resulted in some misclassification due to a one-month overlap between the 2 response options “within the last 6 months” and “between 6 months and a year ago.” Combining these 2 categories to eliminate this misclassification does not change the overall finding of the study that among adults with arthritis, prevalence of receiving HCP counseling about PA in the past year was low (26.4%). Finally, although items used in this study were reviewed by experts for face validity, other forms of validity and test–retest reliability were not established before use in this study.

Strengths of this study were that data were collected from a large sample of US adults and weighted to the 2019 US population. This study informs public health interventions seeking to increase the quantity and effectiveness of HCP counseling about PA among adults with arthritis by better understanding the overall prevalence of HCP counseling about PA for arthritis management, the recency of HCP counseling, the patient-level factors associated with receiving counseling, and the content of the counseling.

The prevalence of HCP counseling about PA reported in this and other research suggests persistent multifaceted challenges to HCP screening, counseling, and recommendation for PA that remain difficult to overcome and may create hesitancy among HCPs. Systemic barriers may include social determinants of health (eg, inconsistent access to affordable health care), health records systems that do not include PA screening, electronic health record noninteroperability, insufficient reimbursement amounts, and poor integration of clinical and community-based PA programs through bi-directional feedback systems. HCP barriers may include insufficient education and skills-based training for PA counseling, lack of time, lack of availability of community-based PA programs, PA programs that are not accessible for patients due to financial or transportation barriers, facilities or equipment that do not accommodate adults with different types of disabilities, concerns that PA programs are not safe or effective, and insufficient awareness of CDC-recognized, arthritis-appropriate, evidence-based interventions (22–24).

Given the unique barriers to PA expressed by adults with arthritis (eg, joint pain, stiffness, fatigue, fear of movement, functional limitations) (25), the importance of HCP PA counseling for adults with arthritis (13,14,16,17), and the many multilevel barriers to assessment, counseling, and recommendation, there are opportunities for increasing HCP awareness about existing, but underutilized, evidence-based tools and programs that can be leveraged to improve PA behaviors among adults with arthritis (26). A National Public Health Agenda for Osteoarthritis 2020 (16) provides a menu of strategies and actions that address some of the gaps and opportunities identified in this study. The Agenda specifically calls for public health action to 1) promote PA as a vital sign, focusing on its benefits in reducing arthritis pain and preventing or managing chronic comorbidities such as diabetes, heart disease, overweight or obesity, depression, and anxiety that frequently co-occur with arthritis and for which PA is a proven strategy for prevention or management; 2) increase awareness about effective community-based PA interventions for adults with arthritis; 3) increase awareness of and facilitate HCP counseling and recommendation behaviors, including integrating training and interventions into the curricula of medical and allied health programs, residencies, and fellowships; and 4) undertake research to inform these and related efforts (16).

Programs such as Exercise is Medicine (www.exerciseismedicine.org), Walk with a Doc (walkwithadoc.org), and Park Rx America (parkrxamerica.org) can be leveraged to increase HCP awareness of the importance of screening, counseling, and recommendation, as well as link providers to tools and resources to facilitate or bolster PA counseling and patient recommendations to community-based PA programs and opportunities (27–29). Some existing resources that clinical and public health professionals can use to enhance HCP counseling about PA among adults with arthritis are 1) the Osteoarthritis Action Alliance OACareTools for HCPs and adults with arthritis (30); 2) Exercise is Medicine Health Care Providers Action Guide for PA counseling and supporting tools (29); 3) Exercise is Medicine training modules for health professionals including a module specifically for osteoarthritis (31); and 4) a brief Medscape continuing medical education module for HCPs to assess their knowledge of evidence-based lifestyle interventions for arthritis (available for free through November 2025) (32). Consistent with the Physical Activity Guidelines for Americans Midcourse Report: Implementation Strategies for Older Adults (23), CDC collaborates with the Osteoarthritis Action Alliance to recognize and promote arthritis-appropriate evidence-based interventions (26,33) that can be offered within a variety of settings (eg, home, health care, community). Health care systems, health professionals, and community organizations may also benefit from joining the Active People, Healthy Nation initiative (34), which is a multisector initiative promoting PA inclusive of all ages and abilities, race or ethnicity, sex or sexual identity, income, and place of residence (eg, urban or rural).

Additionally, more formative research as well as research examining the efficacy and effectiveness of multilevel interventions (eg, systems, policy, provider, and patient) is needed to increase HCP screening, counseling, and recommendation for PA. To date, most research has focused on clinicians/HCPs (24), patients (22), and community health workers (35), with less research exploring the perspectives and experiences of other affected groups. To better understand how these interventions should be structured and delivered, future research might examine the preferences and perspectives of other interested groups, such as payors, health care administrators, health information technology professionals, community-based PA organizations, and caregivers. Finally, dissemination and implementation research aiming to increase the proportion of adults with arthritis that initiate and complete an evidence-based PA program after receiving an HCP recommendation (ie, linkage and retention) is needed.

In conclusion, 3 important lessons were learned from this study for HCPs and public health professionals. First, although over half (54.1%) of adults with arthritis report ever-received HCP counseling about PA, only about 1 in 4 (26.4%) adults with arthritis received PA counseling within the past 12 months. This indicates that arthritis-specific HCP counseling about PA is infrequent, despite most adults with arthritis reporting more than 1 health care appointment within the past 12 months. Second, HCP counseling about PA for arthritis management was more frequent among adults with excess weight and chronic pain, and opportunity exists to increase HCP counseling about PA among adults with arthritis who are not yet experiencing obesity, pain, or other detrimental health outcomes. Third, beyond basic information consistent with the national PA guidelines, most patients are not receiving sufficient actionable information or tangible resources to successfully access, adopt, and sustain PA levels sufficient to manage their arthritis symptoms or meet the PA guideline. Taken together, adoption and dissemination of evidence-based policies and programs facilitating early and effective HCP counseling, clinic-to-community recommendations, and successful patient linkage to and retention in arthritis-appropriate evidence-based PA programs among adults with arthritis remain a public health priority.
